# Bone Health and Risk Factors of Cardiovascular Disease - A Cross-Sectional Study in Healthy Young Adults

**DOI:** 10.1371/journal.pone.0108040

**Published:** 2014-10-13

**Authors:** Satu Pirilä, Mervi Taskinen, Maila Turanlahti, Merja Kajosaari, Outi Mäkitie, Ulla M. Saarinen-Pihkala, Heli Viljakainen

**Affiliations:** 1 Children's Hospital, University of Helsinki and Helsinki University Central Hospital, Helsinki, Finland; 2 Folkhälsan Research Centre, Helsinki, Finland; University of California Davis, United States of America

## Abstract

**Objective:**

Both osteoporosis and cardiovascular disease (CVD) are diseases that comprise a growing medical and economic burden in ageing populations. They share many risk factors, including ageing, low phy-sical activity, and possibly overweight. We aimed to study associations between individual risk factors for CVD and bone mineral density (BMD) and turnover markers (BTMs) in apparently healthy cohort.

**Design:**

A cross-sectional assessment of 155 healthy 32-year-old adults (74 males) was performed for skeletal status, CVD risk factors and lifestyle factors.

**Methods:**

We analysed serum osteocalcin, procollagen I aminoterminal propeptide (P1NP), collagen I carboxy-terminal telopeptide (ICTP) and urine collagen I aminoterminal telopeptide (U-NTX), as well as serum insulin, plasma glucose, triglyceride and HDL-cholesterol levels. BMD, fat and lean mass were asses-sed using DXA scanning. Associations were tested with partial correlations in crude and adjusted mo-dels. Bone status was compared between men with or without metabolic syndrome (defined according to the NCEP-ATPIII criteria) with multivariate analysis.

**Results:**

Osteocalcin and P1NP correlated inversely with insulin (R = −0.243, P = 0.003 and R = −0.187, P = 0.021) and glucose (R = −0.213, P = 0.009 and R = −0.190, P = 0.019), but after controlling for fat mass and lifestyle factors, the associations attenuated with insulin (R = −0.162, P = 0.053 and R = −0.093, P = 0.266) and with glucose (R = −0.099, P = 0.240 and R = −0.133, P = 0.110), respectively. Whole body BMD associated in-versely only with triglycerides in fully adjusted model. In men with metabolic syndrome, whole body BMD, osteocalcin and P1NP were lower compared to healthy men, but these findings disappeared in fully adjusted model.

**Conclusions:**

In young adults, inverse associations between BTM/BMD and risk factors of CVD appeared in crude models, but after adjusting for fat mass, no association continued to be present. In addition to fat mass, lifestyle factors, especially physical activity, modified the associations between CVD and bone charac-teristics. Prospective studies are needed to specify the role of mediators and lifestyle factors in the prevention of CVD and osteoporosis.

## Introduction

Both osteoporosis and cardiovascular disease (CVD) are degenerative diseases that comprise an increasing medical and economic burden in ageing populations. Association between these conditions suggest shared pathophysiology [1], shared risk factors [2] or causality [1]. Understanding these is meaningful for the prevention and treatment of these disorders.

Bone compartment is a part of the body composition with a strong correlation with lean mass. Body composition can be assessed accurately with dual energy x-ray absorptiometry (DXA) and bone mineral density (BMD) surrogates a long-term bone status, while circulating bone turnover markers (BTMs) describe the current bone remodelling phase. Other determinants of BMD include physical activity, smoking and dietary intake of calcium and vitamin D. Central adiposity is a key hallmark in the development of metabolic syndrome, which is characterised by a clustering of risk factors (impaired glucose tolerance, unfavourable lipid profile, elevated blood pressure and low grade inflammation) for CVD and type 2 diabetes [3]. On the other hand, central adiposity is linked to inferior bone quality and altered bone turnover, as assessed in bone biopsies in premenopausal women [4]. An association between metabolic syndrome and osteoporosis is conflicting and biased by age, gender, menopausal status, obesity and ethnicity [5]–[8]. In addition, no proof of a causal relationship between metabolic syndrome and osteoporosis has been reported.

Our aim was, first, to study the associations between individual risk factors of CVD and bone characteristics (BMD and BTMs) in a cohort of 32-year-old apparently healthy men and women. Second, we explored the role of lifestyle factors as modifiers of this association. At the age of 32 years, peak bone mass [9] is still maintained and only a few risk factors for CVD [10] may have emerged, and accordingly, pathophysiology could be explored in an unbiased setting.

## Subjects and Methods

### Ethics Statement

The study was approved by the Ethics Committee of the Helsinki and Uusimaa Hospital District in Helsinki, Finland. Written informed consent was obtained from all participants.

### Subjects

This study cohort was part of a prospective evaluation of the impact of early infant feeding pattern on adult bone health, body composition, metabolic features and cardiovascular health [9]–[11]. The original study cohort consisted of 238 healthy full-term Finnish newborns with a birth weight over 3,000 g, born in 1975 at the Helsinki University Central Hospital [12], [13]. At 32 years of age, all subjects from the original cohort were traced through the Population Registry Centre in Finland and invited for a follow-up assessment during 2006 to 2009. Of the 188 subjects who were reached, 158 (84%) consented to participate and were examined at the outpatient clinic at the Children's Hospital, Helsinki University Central Hospital, Finland. The reason for non-participation was lack of time or interest. Subjects with known type 1 diabetes (n = 3) were excluded from the present study, resulting in a study cohort of 155 adults (74 males and 81 females) (Table 1).

**Table 1:** Characteristics of the study cohort. Means (SD) are given, unless otherwise indicated.

Characteristics	All N = 155		Males N = 74		Females N = 81		P
	Mean	SD	Mean	SD	Mean	SD	
Age at study, (range) yrs	32.5	(31.7–34)	32.4	(31.7–33.9)	32.5	(31.7–34.0)	0.83
Weight, kg	75.2	(14.3)	83.8	(12.3)	67.2	(11.2)	<0.001
Height, m	1.74	(0.09)	1.79	(0.07)	1.68	(0.06)	<0.001
BMI, kg/m^2^	24.8	(3.8)	25.8	(3.3)	24.0	(4.1)	0.003
Waist circumference, cm	83.8	(11.8)	89.9	(10)	78.5	(10)	<0.001
Fat mass,* kg	19.6	(7.4)	18.6	(7.1)	20.5	(7.7)	0.11
Fat,* %	25.6	(7.1)	21.5	(5.5)	29.5	(6.2)	<0.001
Lean mass,* kg	53.8	(11.0)	63.2	(7.1)	45.1	(5.3)	<0.001
Bone mineral density,* g/cm^2^							
Whole body	1.14	(0.09)	1.17	(0.09)	1.12	(0.08)	<0.001
Femoral neck	0.85	(0.12)	0.88	(0.12)	0.82	(0.11)	0.004
Lumbar spine	1.04	(0.12)	1.05	(0.12)	1.04	(0.11)	0.56
Blood pressure,† mm Hg							
Systolic	120.0	(13.6)	126.6	(12.7)	113.8	(11.4)	<0.001
Diastolic	73.5	(8.5)	76.1	(8.8)	71.1	(7.6)	<0.001
Lifestyle factors							
Smoking, cigarettes per day	3.9	(7.3)	5.1	(8.5)	2.9	(5.9)	0.07
Alcohol score‡	3.5	(1.0)	3.74	(1.1)	3.3	(0.9)	0.012
Physical activity score¶	3.2	(0.9)	3.1	(1.0)	3.3	(0.9)	0.14

*measured by DXA.

† mean of two measurements.

‡ Scale 1–6 (1 no alcohol to 6 daily usage).

¶ Scale 1–5 (1 no physical activity to 5 regular exercise at least 3 times a week).

### Anthropometric measurements and body composition

At 32 years of age, all participants underwent a physical examination and an interview by one of the authors (SP). At the same visit, height to the closest 0.1 cm and weight to the closest 0.1 kg were measured in light clothing and without shoes. Body mass index (BMI) was calculated as weight/height^2^ (kg/m^2^). Waist circumference was measured to the closest 0.1 cm using a non-elastic measuring tape. Blood pressure was measured after a 10-minute rest, and the average of two measurements was used in the analyses.

### Biochemical markers

Venous blood was drawn after an overnight fast, and serum samples were assessed for bone turnover markers: osteocalcin, (EIA, The Roche Elecsys 1010/2010,Indianapolis, IN, USA), procollagen type 1 N-terminal propeptide (P1NP) and carboxy-terminal telopeptide of type I collagen (ICTP)(RIA, Orion Diagnostica, Espoo, Finland) and serum insulin (immunofluorometry). A second void urine sample was assessed for N-terminal telopeptide urine collagen I aminoterminal telopeptide (NTX) (EIA, Wampole Laboratories, Princeton, NJ, USA). In addition, plasma glucose, cholesterol, HDL cholesterol, and triglycerides were all measured using a photometric, enzymatic method (Roche Diagnostics).

### Bone mineral density and body composition

BMD for lumbar spine (LS), femoral neck (FN) and whole body (WB) were measured using DXA (Hologic Discovery A, software version 12.4∶3, Waltham, MA, USA). Body composition, including whole body fat and lean mass, was also assessed using DXA. The coefficient of variation (CV) for BMD was 1.6–1.8% and for body composition was 0.7–1.0% [14].

### Covariates

**Figure 1:**
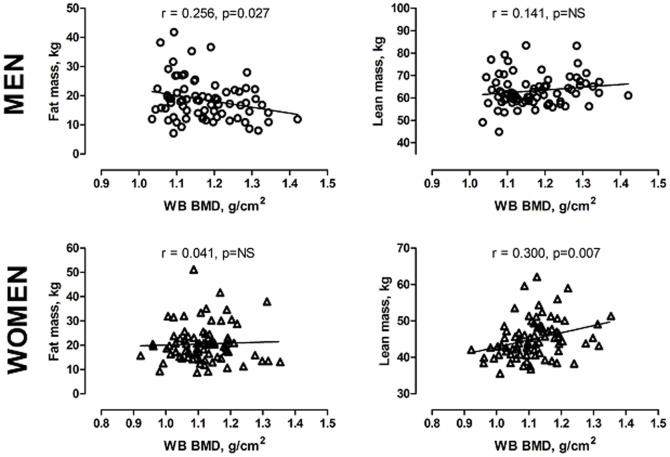
Associations between whole body bone mineral density (BMD), fat mass and lean mass in men (squares) and women (triangles).

Shared determinants for bone strength and cardiovascular risk factors based on the literature were tested as potential confounders. Many variables were dependent on gender, body size and fat mass (Figure 1). Fat mass is expectedly higher with taller persons, and therefore height was also used as a covariate. Lifestyle factors (smoking, alcohol consumption and physical activity) modify both bone remodelling and cardiovascular measures. These were collected as reported earlier [9], [11]. Lifestyle factors were coded for analysis as follows: Physical activity from 1 to 5 (no exercise to regular heavy training at least three times weekly); alcohol consumption from 1 to 6 (no alcohol usage to daily usage). Smoking was defined by the amount of cigarettes smoked per day.

### Statistical analyses

Associations between individual bone and cardiovascular measures were investigated using partial correlations. Four additive models were constructed: gender (Model 1), + height and fat mass (Model 2) and + lifestyle factors of smoking, physical activity and alcohol consumption (Model 3) to explore possible mediators. Continuous variables were used when available. A student's t test was used when comparing differences between two groups (genders) and the chi-square test was utilised for categorised variables. Characterisation of metabolic syndrome was based on plasma glucose concentration, systolic and diastolic blood pressure, HDL cholesterol and triglyceride concentrations, and waist circumference according to the National Cholesterol Education Program Adult Treatment Panel III (NCEP-ATPIII) [3].

Ultimately, multivariate analysis was carried out to evaluate bone measures (BMD, BTMs) between healthy individuals (no-MetS) and those fulfilling the criteria for metabolic syndrome (MetS) [3]; in crude values and fully adjusted models ( =  height, fat mass and life-style factors). In the final analyses, we included only men because of the interaction between gender and outcome variables, and because only two women fulfilled the criteria for metabolic syndrome. A P value of <0.05 was considered significant. All analyses were performed using IBM SPSS Statistics, Version 19.0.

## Results

### Cohort characteristics

All anthropometrics were significantly higher in males compared with females, as expected (Table 1). A higher proportion of males were overweight as compared with females (50% vs. 25%, P = 0.001). Fat percentage was higher in females (P<0.001), but there was no difference in absolute fat mass between the genders. The males had higher systolic and diastolic blood pressure than females (P<0.001). Of the participants, 74% exercised at least twice a week and their alcohol consumption was moderate, up to twice a week in 88%. Altogether 69% did not smoke. However, males smoked more often and consumed more alcohol than females, while no difference was observed in physical activity between the genders.

Males had higher levels of fasting plasma glucose and triglycerides than females (Table 2), but the opposite was observed in plasma HDL-cholesterol (P<0.001). BTMs did not differ between the genders. In males, 12 out of 74 (16.2%) fulfilled the criteria for metabolic syndrome according to NCEP-ATPIII, compared with 2 out of 81 (2.4%) of the females (P = 0.003) (Table 3).

**Table 2:** Bone turnover markers and metabolic characteristics in the study subjects.

Biomarker	All		Males		Females		P
	Mean	SD	Mean	SD	Mean	SD	
Osteocalcin, µg/L	20.5	(9.0)	20.2	(8.8)	21.0	(9.3)	0.59
P1NP, µg/L	50.1	(18.3)	51.2	(15.2)	49.3	(20.9)	0.54
ICTP, µg/L	3.4	(0.9)	3.1	(0.7)	3.7	(0.9)	0.001
NTX, nmol/nmol Crea	48.9	(28.4)	53.0	(33.6)	45.7	(23.0)	0.16
Triglycerides, mmol/L	1.0	(0.5)	1.1	(0.6)	0.8	(0.4)	<0.001
HDL cholesterol, mmol/L	1.6	(0.4)	1.4	(0.3)	1.7	(0.4)	<0.001
Glucose, mmol/L	5.2	(0.5)	5.4	(0.6)	5.0	(0.4)	<0.001
Insulin, mU/L	5.7	(3.0)	6.1	(3.6)	5.3	(2.4)	0.10

Abbreviations: P1NP  =  procollagen type 1 N-terminal propeptide; ICTP  =  carboxy-terminal telopeptide of type I collagen; NTX  =  N-terminal telopeptide urine collagen I aminoterminal telopeptide.

Values are given as mean (standard deviation). P values refer to the difference between males and females.

**Table 3:** Individuals beyond the cut-off limits contributing to metabolic syndrome according to the NCEP-ATPIII criteria [3].

	ALL (N = 155)	Males (n = 74)	Females (N = 81)	P^a^
	N (%)	N (%)	N (%)	
fP-Glucose >5.6 mmol/L	30 (19.5)	25 (33.8)	5 (6.3)	<0.001
fP-Triglycerides ≥1.7 mmol/L	11 (7.1)	9 (12.1)	2 (2.5)	0.027
fP-HDL ≤1.0*/1.3† mmol/L	12 (7.8)	7 (9.5)	5 (6.3)	0.55
Waist circumference ≥102*/88† cm	25 (16.2)	10 (13.5)	15 (18.8)	0.51
SBP >130 and/or DBP >85 mm Hg	38 (24.7)	31 (41.9)	7 (8.8)	<0.001
≥3 NCEP-ATPIII criteria	14 (8.9)	12 (15.8)	2 (2.4)	0.003

Abbreviations: fP, fasting plasma; SBP, systolic blood pressure; DBP, diastolic blood pressure; NCEP-ATPIII, National Cholesterol Education Program Adult Treatment Panel III.

a P values calculated between the genders using Chi-square test;

*male;

† female.

BMI correlated positively with BMD at all measured sites. The detailed body composition assessed by DXA revealed that fat percentage and fat mass had an inverse correlation with BMD, whereas a positive correlation was observed between lean mass and BMD (data not shown). The associations between whole body BMD and body composition by gender are shown in Figure 1. In males, an inverse association was found between whole body BMD and fat mass (R = −0.255, P = 0.027), but no correlation between BMD and lean mass. In contrast, in females, BMD associated with lean mass (R = 0.300, P = 0.007) but not with fat mass.

### Correlations of bone turnover markers and BMD with the characteristics of metabolic syndrome

The data on crude and adjusted partial correlation between BTMs/BMD and the individual risk factors of CVD are presented in Table 4. Bone formation markers, osteocalcin and P1NP, correlated inversely with plasma glucose (R = −0.21, P = 0.003; R = −0.19, P = 0.021) and with serum insulin levels (R = −0.24, P = 0.003; R = −0.19, P = 0.021), respectively. The correlation remained after adjustment for gender, height and fat mass, except for P1NP and insulin, which was attenuated after controlling for fat mass. In the fully adjusted model 3, including lifestyle factors, the associations disappeared signifying that the association between osteocalcin and glucose/insulin is mediated by lifestyle factors.

**Table 4:** Partial correlation coefficients (R) between bone turnover markers/bone mineral density and fasting insulin/individual characteristics of metabolic syndrome defined by NCEP ATPIII.

	Models a	OC		P1NP		ICTP		NTX		LS BMD		FN BMD		WB BMD	
		R*	P	R*	P	R*	P	R*	P	R*	P	R*	P	R*	P
Insulin	Crude	−0.243	0.003	−0.187	0.021	0.025	0.787	−0.111	0.223	−0.011	0.891	0.063	0.439	−0.067	0.407
	Model 1	−0.239	0.003	−0.195	0.016	0.071	0.459	−0.130	0.156	−0.018	0.825	0.022	0.784	−0.123	0.127
	Model 2	−0.168	0.043	−0.108	0.190	−0.034	0.718	−0.053	0.572	−0.097	0.236	−0.058	0.477	−0.084	0.305
	Model 3	−0.162	0.053	−0.093	0.266	−0.040	0.675	−0.056	0.556	−0.065	0.433	−0.089	0.289	−0.075	0.364
Glucose	Crude	−0.213	0.009	−0.190	0.019	−0.232	0.011	−0.022	0.815	0.068	0.405	0.069	0.395	0.078	0.337
	Model 1	−0.211	0.010	−0.228	0.005	−0.124	0.182	−0.079	0.394	0.053	0.517	−0.063	0.118	−0.075	0.351
	Model 2	−0.152	0.066	−0.179	0.029	−0.189	0.044	−0.025	0.789	0.033	0.689	−0.097	0.235	−0.018	0.828
	Model 3	−0.099	0.240	−0.133	0.110	−0.169	0.076	−0.017	0.861	0.032	0.696	−0.114	0.176	−0.017	0.839
Triglycerides	Crude	−0.134	0.103	−0.113	0.164	−0.045	0.626	−0.067	0.470	−0.089	0.273	0.016	0.849	−0.109	0.181
	Model 1	−0.126	0.126	−0.136	0.097	0.054	0.565	−0.112	0.224	−0.108	0.189	−0.083	0.312	−0.228	0.005
	Model 2	−0.037	0.656	−0.048	0.562	−0.038	0.690	−0.041	0.660	−0.185	0.024	−0.165	0.044	−0.210	0.010
	Model 3	0.005	0.949	0.000	0.998	−0.014	0.886	−0.029	0.762	−0.181	0.028	−0.158	0.060	−0.204	0.014
Waist circumference	Crude	−0.153	0.062	−0.077	0.348	−0.031	0.738	−0.088	0.335	0.160	0.048	0.266	0.001	0.106	0.194
	Model 1	−0.148	0.073	−0.112	0.172	0.136	0.142	−0.169	0.065	0.156	0.054	0.114	0.076	−0.060	0.457
	Model 2	0.042	0.617	0.106	0.199	−0.013	0.888	−0.069	0.461	0.158	0.054	0.104	0.207	0.064	0.436
	Model 3	0.069	0.414	0.138	0.097	0.006	0951	−0.057	0.544	0.151	0.068	0.097	0.247	0.065	0.431
HDL	Crude	0.111	0.175	0.024	0.764	0.001	0.995	0.103	0.259	−0.025	0.754	0.224	0.005	−0.107	0.187
	Model 1	0.100	0.227	0.045	0.584	−0.131	0.160	0.164	0.073	−0.008	0.927	−0.121	0.136	0.100	0.221
	Model 2	0.056	0.504	−0.012	0.888	−0.086	0.365	0.131	0.159	0.031	0.708	−0.086	0.296	0.002	0.979
	Model 3	0.059	0.485	−0.044	0.596	−0.109	0.255	0.107	0.256	0.012	0.889	−0.081	0.332	−0.015	0.855
Systolic BP	Crude	−0.129	0.118	−0.080	0.329	−0.086	0.356	0.046	0.613	0.056	0.492	0.079	0.332	0.115	0.156
	Model 1	−0.119	0.150	−0.113	0.165	0.069	0.463	−0.012	0.899	0.038	0.642	−0.075	0.361	−0.043	0.600
	Model 2	−0.057	0.495	−0.056	0.499	0.016	0.866	0.044	0.635	0.012	0.885	−0.115	0.162	−0.004	0.959
	Model 3	−0.027	0.750	−0.030	0.720	0.027	0.778	0.047	0.623	0.016	0.845	−0.119	0.154	−0.003	0.972
Diastolic BP	Crude	−0.109	0.185	−0.042	0.606	0.002	0.983	0.124	0.174	0.009	0.914	−0.047	0.565	0.018	0.823
	Model 1	−0.099	0.232	−0.057	0.484	0.100	0.281	0.094	0.308	−0.005	0.948	−0.148	0.069	−0.083	0.308
	Model 2	−0.056	0.500	−0.015	0.856	0.065	0.491	0.136	0.144	−0.026	0.750	−0.178	0.029	−0.060	0.463
	Model 3	−0.006	0.940	0.020	0.813	0.086	0.372	0.137	0.146	−0.042	0.610	−0.133	0.111	−0.069	0.408

Abbreviations: OC, osteocalcin; P1NP, procollagen type 1 N-terminal propeptide; ICTP, carboxy-terminal telopeptide of type I collagen; NTX, N-terminal telopeptide urine collagen I aminoterminal telopeptide; BMD, bone mineral density; LS, lumbar spine; FN, femoral neck; WB whole body; HDL, high density lipoprotein cholesterol; BP, blood pressure.

a Crude values and additional models: Model 1: + gender, Model 2: + fat mass and Model 3: + lifestyle factors (smoking, alcohol consumption and current physical activity).

*R, Correlation coefficients.

Neither bone resorption markers (ICTP and urine NTX) nor BMD (at FN, LS and WB) correlated with serum insulin levels (Table 4). Urine NTX did not correlate with any of the individual characteristics of metabolic syndrome. In contrast, plasma ICTP showed a negative correlation with plasma glucose level in the crude and adjusted models (R = −0.189, P = 0.044).

LS BMD (R = 0.160, P = 0.048) and FN BMD (R = 0.197, P = 0.014) correlated with waist circumference, and FN BMD correlated with plasma HDL-cholesterol level (R = −0.173, P = 0.032) in the crude models. These correlations disappeared after adjustment for gender. A negative correlation was uncovered between FN BMD (R = −0.167, P = 0.042) and LS BMD (R = −0.185, P = 0.024) with plasma triglyceride level after adjustment for fat mass. Correspondingly, an association between WB BMD and plasma triglycerides emerged after controlling for gender (R = −0.228, P = 0.005) and remained after additional adjustments for height (R = −0.241, P = 0.003) and fat mass (R = −0.210, P = 0.010) (Table 4). The correlation did not change after additional adjustment for lifestyle factors.

### Bone turnover markers/BMD and the presence of metabolic syndrome

Using multivariate analysis, the differences in BTMs and BMD were evaluated between healthy subjects (no-MetS) and those with an increased risk of metabolic syndrome (MetS  =  NCEP ATPIII criteria ≥3 present). Because of the interaction between gender and the outcome variables, the analyses were performed separately in men and women. In the end, the analysis was performed only in men because of the low number (N = 2) of women fulfilling the criteria for metabolic syndrome.

**Figure 2:**
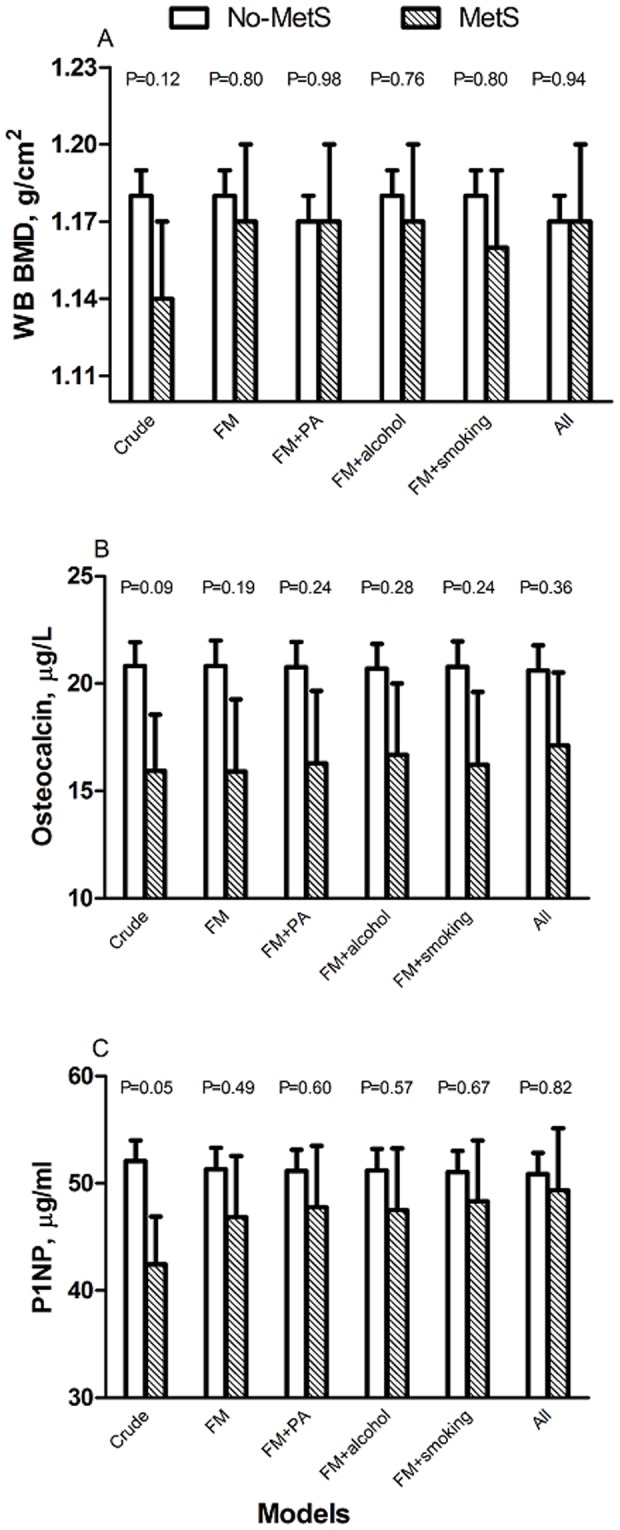
Differences in whole body BMD, osteocalcin and P1NP between healthy men (no-MetS) and those with metabolic syndrome (MetS) (NCEP ATPIII >3), with crude values and after adjusted for height (H) and fat mass (FM), and then additionally adjusted separately for each lifestyle factor; physical activity (PA), alcohol consumption and smoking and final adjustment for all confounding factors (ALL).

Crude analyses showed that the concentrations of P1NP and osteocalcin were slightly higher in the no-MetS group compared with the MetS group, (P = 0.051 and P = 0.089, respectively) (Figure 2). The difference between the groups regarding P1NP and osteocalcin was flattened after adjusting for fat mass (P = 0.50 and 0.22, respectively), proposing fat mass as a possible mediator. After adjustment for lifestyle factors, the difference was attenuated and modified regarding both bone formation markers (P = 0.82 and P = 0.36, respectively), which suggests that these covariates mediate the association between bone formation and metabolic syndrome. Other bone turnover markers did not differ between participants with and without metabolic syndrome in any of the models (Data not shown).

In crude analyses, whole body BMD tended to be lower in men with MetS compared with those without MetS (P = 0.117). There were no differences in other BMD sites (data not shown). Adjustment for fat mass and height attenuated the difference between the groups (P = 0.80), and again lifestyle factors further modified the association, suggesting that these factors were stronger determinants for BMD and metabolic syndrome (P = 0.94) (Figure 2).

## Discussion

We observed inverse associations between bone health and individual risk factors of CVD in the present cross-sectional study: low concentration of both bone formation markers, osteocalcin and P1NP were accompanied with elevated fasting insulin and glucose levels in healthy adults at 32 years of age. The final analyses showed that men with metabolic syndrome had lower P1NP concentrations compared with healthy subjects, and a similar, but non-significant, trend was observed for osteocalcin. Parallel, but non-significant, findings were seen for whole body BMD, which was lower in men with metabolic syndrome than in healthy men. Differences between the groups were attenuated after adjustments for the confounders, emphasising the roles of fat mass and lifestyle factors (physical activity, smoking and alcohol consumption).

The role of osteocalcin in glucose metabolism and as a link between bone and adipose tissue has been under active research. In an animal model, osteocalcin deficiency was associated with glucose intolerance, reduced insulin sensitivity and increased adiposity [15]. Furthermore, the administration of recombinant osteocalcin improved the measures of glucose and insulin metabolism in wild-type animals [16]. Corresponding human studies have been conducted mostly in patients with T2DM [17], metabolic syndrome [18], [19], coronary heart disease [18], osteoporosis therapy [20] or hypertension [19], and these have witnessed low osteocalcin to associate with higher fasting plasma insulin, glucose and triglyceride levels without proof of causality. Studies assessing the role of osteocalcin in metabolically healthy subjects are scarce. Polgreen et al. (2012) reported inverse relationships between osteocalcin and BMI, waist circumference, systolic blood pressure and insulin resistance in young healthy adolescents and adults at a mean age of 19 years [21]. Their findings were attenuated after further adjustments for BMI, also suggesting that BMI was the mediator. Our findings are in accordance with these observations, and confirm inverse associations between osteocalcin and risk factors of CVD in apparently healthy young adults. Furthermore, we found that similar to osteocalcin, P1NP correlated inversely with both glucose and insulin. On the other hand, bone resorption markers were not associated with any risk factors of CVD.

The aspect of whether or not body fat is beneficial for bone is controversial. It has been generally accepted that overweight and high BMI protect against osteoporosis. This might not apply to all age groups, beyond the range of normal weight, or for both genders [22]–[24]. The balance between bone formation and resorption varies in childhood, in young adulthood and in advanced age/elderly subjects, which might explain the controversial results. Higher BMI was found to protect against osteoporotic and other fractures, especially in lean subjects, however, after controlling for BMD, the risk ratio became non-significant in all BMI categories in a large meta-analysis of over 60,000 participants from 12 different prospective cohorts [25]. A study on growing teenagers by Lawlor et al. reported a positive association between fat mass and bone mass in adolescents aged 15 years, and concluded that the finding was not mediated through fasting glucose or lipids [8]. In the present study, an inverse association was observed between fat mass and BMD in males. Corresponding results have been reported in other cohorts; Hsu et al. [26] reported that higher fat percentage was associated with lower bone mineral content (BMC) in Chinese men and women aged 25–64 years. Confounders taken into account included physical activity, weight and years from menopause. Similar to us, they noticed that plasma lipids were also negatively associated with BMC [26]. In another study, African-American female college students from the USA with a high percentage body fat (>32%) had lower cortical bone strength at 19 year of age than those with a normal fat percentage [27]. Our findings suggest that excessive fat contributes to metabolic disturbances such as insulin resistance, elevated blood pressure and triglycerides, which initially appear to be inversely related to skeletal parameters, at least in men. A deeper examination revealed that these correlations were mediated by fat mass and lifestyle factors. Thus, no associations were left between bone and glucose metabolism after taking into account fat mass, physical activity, smoking and alcohol consumption. In addition, a deeper examination uncovered independent risk factors for BMD of which triglycerides, but less likely diastolic blood pressure, appeared consistently and inversely related to BMD. In accordance with our finding, a Korean study reported high triglyceride concentration to associate inversely with femoral neck BMD after adjustments in men aged 48 years [7].

Metabolic syndrome comprises a cluster of risk factors for CVD, and in the ultimate analysis we compared BTMs and BMDs between men with vs. without metabolic syndrome. In crude analyses, bone formation markers and WB BMD tended to be lower in men with metabolic syndrome compared with those without metabolic syndrome, but the differences were attenuated by fat mass and lifestyle factors. Parallel to our findings, BMD was lower in premenopausal and postmenopausal Korean women with metabolic syndrome compared to those without metabolic syndrome [5]. The explanation offered by Jeon et al. is the higher inflammation status among those with metabolic syndrome, as measured by C-reactive protein, suggesting that obesity and metabolic syndrome maintain inflammation that activates bone resorption [5]. We are not arguing with this explanation, but we would like to know whether the association would remain after adjusting for fat mass. In addition, smoking, excessive alcohol consumption [28] and low physical activity [29] are all related to an elevated risk of these non-communicative diseases. Later in life, especially in women, diminishing levels of oestrogen impair bone health and increase the risk of CVD. Nutritional habits can influence the subsequent risk of developing metabolic syndrome, and building and maintaining stronger bones.

No conclusion about causality between bone health and metabolic syndrome could be drawn in our study. For that purpose, follow-up studies are needed. Another limitation of our study, related to the small study cohort that consisted of single age group and young age of the subjects, was the low number of women who fulfilled the criteria of metabolic syndrome, prohibiting analysis in women. In addition, it may be that our findings cannot be generalised to other ethnic populations, as all of the subjects were white Caucasians. However, the homogeneity of our study population in respect of ethnicity, age and lack of cardiovascular morbidity can be considered as strength. Furthermore, a DXA-derived assessment of body composition is a more reliable measure of body adiposity than the generally used BMI or weight. For bone health, we broadly used both BTMs and BMD to evaluate the associations, and a wide range of potential confounding factors were included in the analysis. Young adulthood is a novel target group for studying the association between bone health and risk of CVD while BMD is at its peak level and the risk factors of CVD are emerging.

Our most important conclusion is that the inverse association between bone formation markers (osteocalcin, P1NP) and fasting glucose and insulin in our cohort of young adults was mediated mainly by fat mass, while triglycerides remained, independently of fat mass, inversely associated to BMD. Men with metabolic syndrome had somewhat lower BTMs and WB BMD compared with men without metabolic syndrome, but the differences disappeared when fat mass was introduced: this reinforce the role of fat mass as a mediator. Future studies in older age groups are warranted to confirm our findings. Especially prospective studies are needed to further study the pathophysiology between CVD and osteoporosis, to address the causal relationships between bone and energy metabolism and to specify the role of mediators, lifestyle factors, in the prevention of osteoporosis and CVD.
